# Healthcare Contacts after Myocardial Infarction According to Mental Health and Socioeconomic Position: A Population-Based Cohort Study

**DOI:** 10.1371/journal.pone.0134557

**Published:** 2015-07-30

**Authors:** Tine Jepsen Nielsen, Mogens Vestergaard, Morten Fenger-Grøn, Bo Christensen, Karen Kjær Larsen

**Affiliations:** 1 Mental Health in Primary Care (MEPRICA), Research Unit for General Practice, Department of Public Health, Aarhus University, Aarhus, Denmark; 2 Section for General Medical Practice, Department of Public Health, Aarhus University, Aarhus, Denmark; Medical University of Vienna, AUSTRIA

## Abstract

**Objective:**

To examine the long-term use of healthcare contacts to general practice (GP) and hospital after a first-time myocardial infarction (MI) according to mental health and socioeconomic position.

**Methods:**

Population-based cohort study of all patients discharged with first-time MI in the Central Denmark Region in 2009 (n=908) using questionnaires and nationwide registers. We estimated adjusted incidence rates and incidence rate ratios (IRR) for GP and hospital contacts according to depressive and anxiety symptoms, educational level and cohabitation status.

**Results:**

During the 24-month period after the MI, patients with anxiety symptoms had 24% more GP contacts (adjusted IRR 1.24, 95% confidence interval (CI) 1.12–1.36) than patients with no anxiety symptoms. In contrast, patients with depressive symptoms (1.05, 0.94–1.16) and with short and medium education (<10 years: 0.96, 0.84–1.08; 10–12 years: 0.91, 0.80–1.03) and patients living alone (0.95, 0.87–1.04) had the same number of GP contacts as their counterparts (patients with no depressive symptoms, with long education [>12 years] and patients living with a partner). During the first 6 months after the MI, patients living alone had 13% fewer hospital contacts (0.87, 0.77–0.99), patients with short education had 16% fewer hospital contacts (<10 years: 0.84, 0.72–0.98) and patients with anxiety symptoms had 27% fewer hospital contacts (0.73, 0.62–0.86) than their counterparts. In contrast, patients with depressive symptoms (0.92, 0.77–1.10) and medium education (10–12 years: 1.05, 0.91–1.22) had the same number of hospital contacts as their counterparts.

**Conclusions:**

This study indicates that patients with depressive symptoms, short and medium education and patients living alone have a lower long-term use of healthcare contacts following MI than patients without these risk factors. Patients with depressive symptoms and low socioeconomic position would be expected to have a higher need of healthcare after MI as they have a poorer prognosis.

## Introduction

Mounting evidence suggests that mental health [1–3] and socioeconomic position [4–6] play an important role for the prognosis after myocardial infarction (MI). The underlying mechanisms remain poorly understood even if several possible pathways have been suggested. A biological pathway suggests that depressive and anxiety symptoms may negatively affect mortality through increased inflammation, decreased heart rate variability and endothelial dysfunction. [7,8] A behavioural pathway suggests that depressive symptoms, living alone and short education may negatively affect mortality through low adherence to recommended lifestyle [9–12] and secondary prophylactic medication. [9,11] The behavioural pathway may also include low use of healthcare after MI among patients with poor mental health and low socioeconomic position. It is well-documented that cardiac rehabilitation programmes after MI reduce mortality through risk factor management. [13,14] However, in short-term cardiac rehabilitation programmes, it has been difficult to recruit patients with poor mental health and low socioeconomic position. [15–18] No study has evaluated the long-term overall use of healthcare after MI according to mental health and socioeconomic position.

We aimed to examine the long-term use of general practice (GP) and hospital after a first-time MI according to depressive and anxiety symptoms, educational level and cohabitation status.

## Methods

We conducted a population-based cohort study comprising people living in the Central Denmark Region (1,250,000 inhabitants) with a first-time MI. The cohort has been used in several other studies, [2,3,19–21] and the establishment of the cohort is described in Larsen et al. [19]In this study we used the same baseline patient characteristic data as in the previous studies, but in addition we included new data on healthcare contacts.

### Patients and participants

We consecutively invited all patients above 18 years who were discharged with first-time MI from 1 January 2009 to 31 December 2009 and living in the Central Denmark Region. The patients were identified from the Danish National Hospital Register (DNHR) [22] that stores information on discharge diagnoses classified according to the International Classification of Diseases (ICD-10) on all patients treated as in- or outpatients at any Danish hospital. We received data on patients discharged with MI (code I21) from the DNHR on a monthly basis. To identify incident cases, we excluded those who had been discharged with MI between 1994 and 2008 according to the DNHR. There were no other exclusion criteria. Information on name, current address and vital status was obtained from the Civil Registration System, [23] which also provided the unique personal identification number used to link data between registers and questionnaires.

A pilot-tested hard-copy questionnaire was sent to all eligible participants 12 to 14 weeks after their discharge from hospital; and non-responders received two reminders. The study was approved by the Danish Data Protection Agency (J.no. 2009-41-3018) and the Scientific Research Evaluation Committee of the Danish Academy of General Practitioners (ref. no. 03–2009), and written informed consent was obtained from all participants.

### Healthcare contacts

In Denmark, healthcare services are tax-financed and available to the patients free of charge. The general practitioners act as gatekeepers and as first-line providers in the sense that referral from a general practitioner is always required to initiate in- and outpatient hospital treatment (except for emergency treatment). [24] The long-term chronic care management of MI and depression treatment primarily takes place in GP.

#### General practice

All GP services in daytime and out-of-hours (OOH) provided to citizens in Denmark are registered prospectively with specific codes in the Danish National Health Service Register. [25] The registration is based on a fee-for-service remuneration to the provider and is thus considered very accurate. [25] We included all contacts in daytime (consultations, home visits, e-mail and telephone consultations) and OOH (consultations, home visits and telephone consultations).

#### Hospital

We included all somatic outpatient visits, hospital admissions and emergency department visits based on data from the DNHR [22].

We collected information on healthcare contacts from 12 months before to 24 months after the MI. For all contact types (GP contact in daytime, OOH contact, outpatient visit, hospital admission, emergency department visit), only one contact was included per day per patient.

### Participant characteristics

#### Socioeconomics

Data on age (<60, 60–80, >80 years) at MI and sex were obtained from the Civil Registration System. [23] Data on education (<10 years: primary and lower secondary school; 10–12 years: vocational education and upper secondary school; >12 years: short, medium, and long-term higher education) and cohabitation status (cohabiting, living alone) from the year before MI (2008) were retrieved from the Danish Integrated Database for Labour Market Research. [26]

#### Depressive and anxiety symptoms

We assessed depressive and anxiety symptoms using the Hospital Anxiety and Depression Scale (HADS). [27] The participants were categorised as having depressive or anxiety symptoms if they had a score of ≥8 on the HADS-D or the HADS-A scale. The HADS is designed to be valid in clinical populations with symptoms of physical disease, and it hence leaves out items that may be endorsed by physical rather than mental states. [27,28] The HADS has previously been validated in MI patients and has proven to have satisfactory reliability (HADS-D and HADS-A Cronbach’s α≈0.80). [29,30] Among MI patients, a HADS-D≥8 identified possible cases of depression with a sensitivity of 65% and a specificity of 90% (compared with a diagnosis of depression based on a structured clinical interview for DSM-IV). [31] Among acute coronary syndrome patients, a HADS-A≥8 identified possible cases of anxiety with a sensitivity of 91% and a specificity of 61% (compared with a diagnosis of generalised anxiety disorder based on a structured clinical interview for DSM-IV). [32]

#### Dyspnoea score

In epidemiological studies, a score ≥3 on the Medical Research Council dyspnoea scale [33] (i.e. walks slower than contemporaries on level ground because of breathlessness, or has to stop for breath when walking at own pace, or worse) has been shown to provide a simple and valid method for predicting all-cause [34,35] and cardiovascular mortality. [35]

#### Comorbidity

The DNHR [22] provided information on stroke (ICD-10: I61, I63, I64) and heart failure (ICD-10: I11.0, I13.0, I13.2, I42.0, I42.6, I42.7, I42.9, I50.0, I50.1, I50.9) from 1994 to 2008. The Danish National Diabetes Register provided information on diabetes from 1990 to 2008 according to an algorithm developed on the basis of information from four nationwide registers. [36]

#### Drug prescription data

These were obtained from the prescription database. [37] Data on aspirin (ATC: B01AC06), clopidogrel (ATC: B01AC04), statins (ATC: C10AA), β-blockers (ATC: C07), ACE-inhibitors/angiotensin 2 receptor blockers (ATC: C09), and antidepressants (ATC: N06A) were collected. We calculated whether the participant had tablets available on the day that we sent the questionnaire (the number of tablets on the last redeemed prescription before the questionnaire was sent ≥ the number of days to the questionnaire was sent) and defined the participant as ‘receiving treatment’ if tablets were available. We defined the participant as ‘receiving secondary prophylactic medication’ if the participant was receiving treatment with three or more of the following drugs: aspirin, clopidogrel, statins and β-blockers.

#### Health behaviour

Data on smoking and physical activity were self-reported in the questionnaire. [3]

### Statistical analysis

For all patients discharged with a first-time MI, we estimated monthly contact rates to GP and hospital from 12 months before to 24 months after the MI. For all patients, we further calculated average contact rates per month for the periods 12 months before, 2–24 months after (for GP contacts), 2–6 months after (for hospital contacts) and 7–24 months after (for hospital contacts) the first-time MI.

For participants (those who returned the questionnaire), we estimated 3-monthly contact rates to GP and hospital according to depressive symptoms, anxiety symptoms, educational level and cohabitation status. We calculated unadjusted and adjusted contact rates, and adjusted incidence rate ratios (IRRs) per 3 months from 12 months before to 24 months after the MI. We further calculated adjusted IRRs for the first 6 months after (for hospital contacts) and the 24 month-period after (for GP contacts) the MI. Adjustment were carried out by standardising to mean values of depressive symptoms, anxiety symptoms, educational level, cohabitation status, dyspnoea score, comorbidity, sex and age. In sub-analyses of all patients discharged with a first-time MI, we estimated unadjusted 3-monthly contact rates to GP according to the available patient characteristics (cohabitation status and educational level).

A negative binomial model was applied for the calculation of estimates and corresponding 95% confidence intervals (CI) for incidence rates and IRRs. Robust variance estimation with clustering at patient level was used to account for heterogeneity between subjects. To account for differences in follow-up time, log-transformed risk time was included in the model with the regression parameter restricted to 1. To account for differences in follow-up, censoring was done when a person died or emigrated. The index date and the day prior to the index date were contained in the first period after the MI diagnosis to allow for the possibility of delay in the administrative coding of the MI diagnosis. No variable had more than 3.4% missing data, and analyses were performed on complete data only.

## Results

### Patients and participants

**Fig 1:**
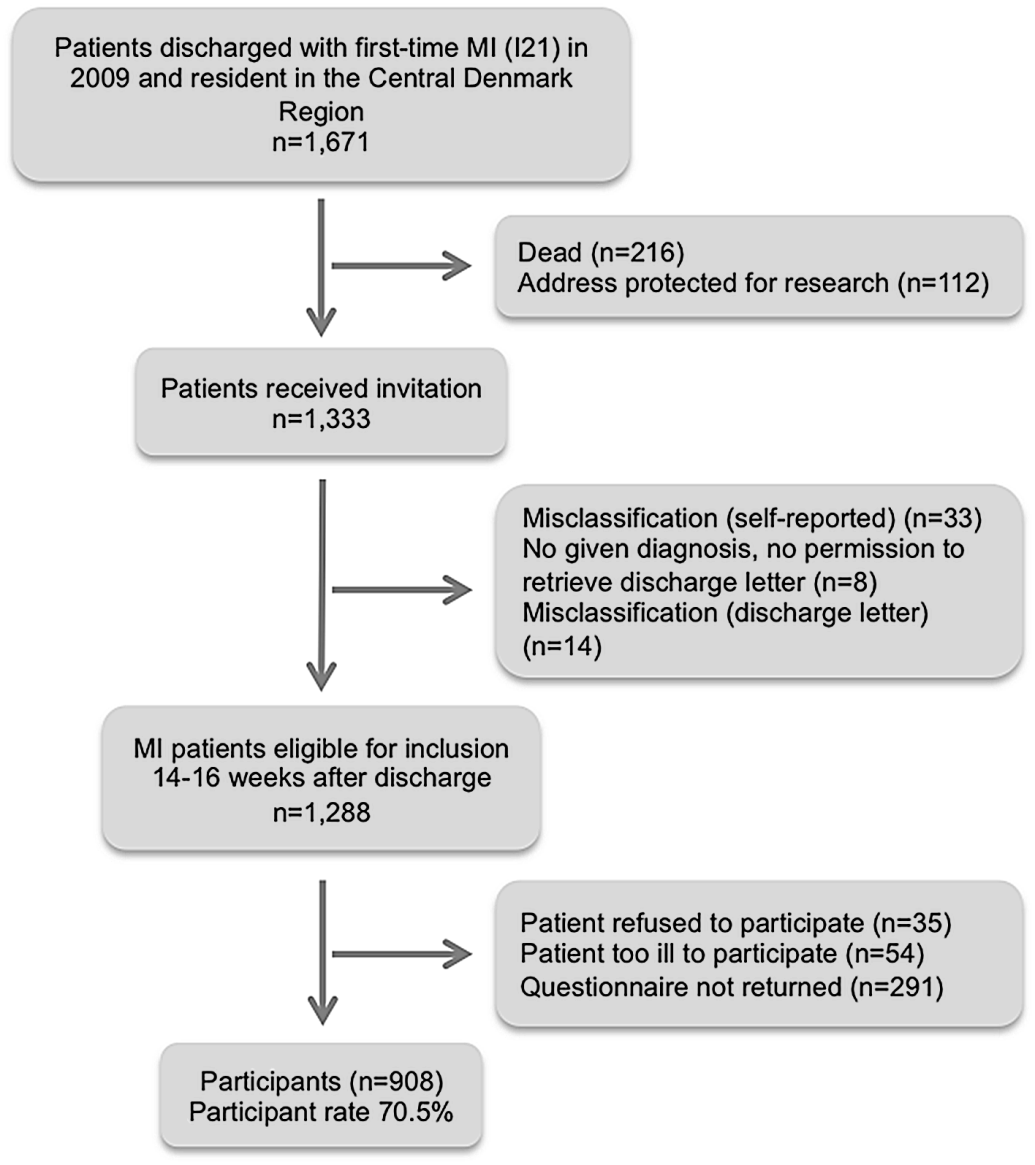
Flowchart for cohort.

A total of 1,671 patients were discharged with a first-time MI in 2009 in the Central Denmark Region. Among them, 1,288 were eligible for inclusion after 14–16 weeks, and 908 (70.5%) returned the questionnaire and were included as participants (Fig 1). Participants (n = 908) were more likely to be men, younger, have a longer education, live with a partner, and not have a comorbid condition than were both the entire group of patients discharged with first-time MI (n = 1,671) and the eligible patients (n = 1,288) (Table 1).

**Table 1:** Characteristics of patients and participants.

Characteristics	All MI patients (n = 1,671)^a^	Eligible patients (n = 1,288)^a^	Participants (n = 908)^a^
Male sex, No. (%)	1056 (63.2)	834 (64.8)	626 (68.9)
Age, mean (SD)	69.7 (13.6)	68.5 (12.9)	67.1 (11.7)
Education, No. (%)^b^			
<10 years	764 (50.5)	586 (48.6)	398 (45.4)
10–12 years	566 (37.4)	469 (38.9)	362 (41.3)
>12 years	183 (12.1)	150 (12.4)	117 (13.3)
Cohabitation status, living alone, No. (%)^b^	709 (43.0)	508 (39.4)	287 (31.6)
Depressive symptoms, HADS-D ≥8, No. (%)^c^	-	-	167 (18.6)
Anxiety symptoms, HADS-A ≥8, No. (%)^c^	-	-	211 (23.5)
Dyspnoea score ≥3, No. (%)^c^	-	-	184 (20.5)
Comorbidity, No. (%)^d^	477 (28.5)	338 (26.2)	195 (21.5)

^a^Numbers may not sum to their respective totals due to missing data.

^b^Information collected the year before MI (in 2008).

^c^Information collected three months after MI.

^d^Information collected at the time of MI.

**Fig 2:**
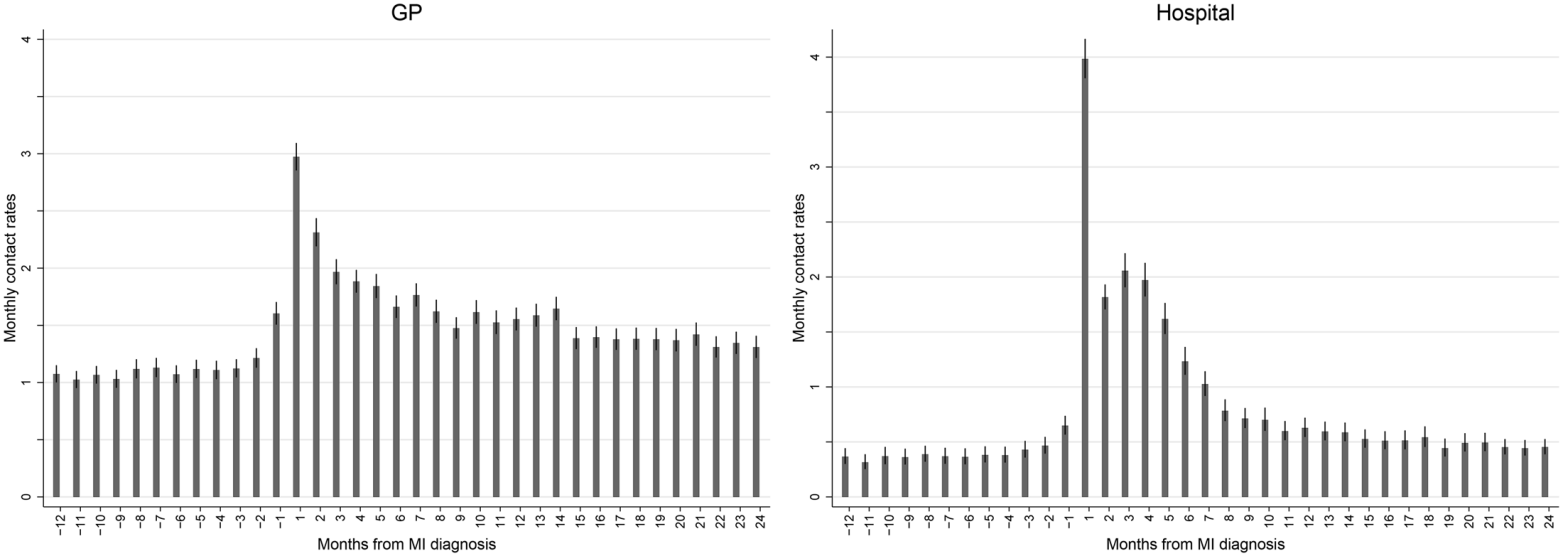
Monthly contact rates (with 95% CI) to general practice and hospital for all MI patients (n = 1,671) before and after first-time MI. The date of MI is contained in the first period after the MI diagnosis.

Before the MI, the patients (n = 1,671) had a stable level of 1.14 (95% CI 1.09–1.19) GP contacts per month. In the first month after the MI, a substantial increase to 2.97 (2.86–3.09) was observed, and during the 2-24-month period after the MI the rate of GP contacts stabilised around 1.58 (1.53–1.64) GP contacts per month (Fig 2).

Before the MI, the patients had a stable level of 0.40 (95% CI 0.35–0.46) hospital contacts per month. In the first month after the MI, a substantial increase to 3.98 (3.81–4.16) was observed. In the hospital rehabilitation period (2–6 months after the MI), they had 1.74 (1.65–1.84) contacts, and the level of contacts subsequently stabilised around 0.59 (0.53–0.65) hospital contacts per month (Fig 2).

### GP contacts

**Fig 3:**
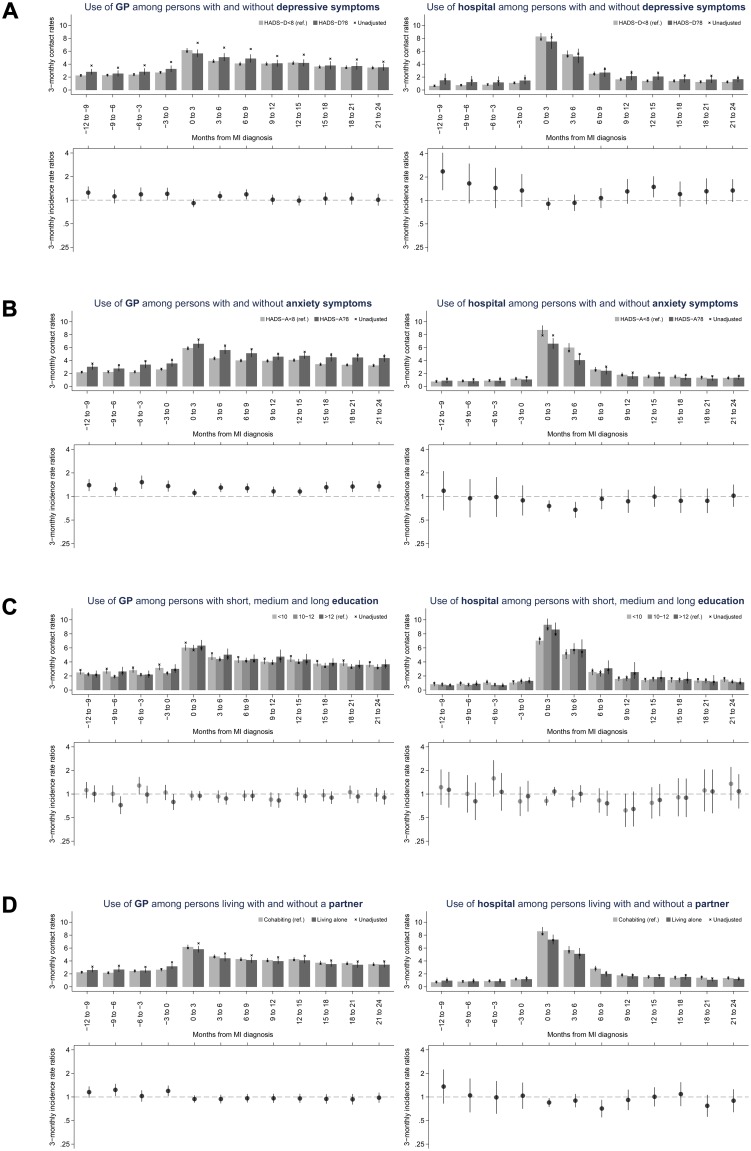
Use of general practice and hospital before and after first-time MI per 3-months periods according to mental health and socioeconomic position. Upper part: Unadjusted and adjusted (with 95% CI) contact rates. Lower part: Adjusted incidence rate ratios (with 95% CI). The date of MI is contained in the first period after the MI diagnosis.

During the 24 months following the MI, patients with anxiety symptoms had 24% more GP contacts (adjusted IRR 1.24, 95% CI 1.12–1.36) than patients with no anxiety symptoms. In contrast, patients with depressive symptoms (1.05, 0.94–1.16), short and medium education (<10 years: 0.96, 0.84–1.08; 10–12 years: 0.91, 0.80–1.03) and patients living alone (0.95, 0.87–1.04) had a number of GP contacts similar to that of their counterparts (patients with no depressive symptoms, with long education (>12 years) and patients living with a partner) (Fig 3).

### Hospital contacts

During the first 6 months after the MI, the hospital contact rate was the same among patients with depressive symptoms (adjusted IRR 0.92, 95% CI 0.77–1.10) and patients with medium education (10–12 years: 1.05, 0.91–1.22) as among their counterparts. Inversely, the hospital contact rate was lower among patients living alone (0.87, 0.77–0.99) and among patients with short education (<10 years: 0.84, 0.72–0.98) and with anxiety symptoms (0.73, 0.62–0.86) than among their counterparts (Fig 3).

### Drug prescriptions and health behaviour

Patients with depressive symptoms were less likely to receive secondary prophylactic medication than patients without depressive symptoms (65.9% vs. 73.7%, p = 0.041). Patients with short education (79.9% vs. 87.6% and 85.5%, p = 0.014) and patients living alone (77.0% vs. 86.2%, p = 0.001) were less likely to receive statin treatment compared to their counterparts. Among patients with depressive symptoms, 22.2% received antidepressants, whereas 19.0% of patients with anxiety symptoms and 15.0% of patients living alone received antidepressants. Patients with depressive symptoms and anxiety symptoms were physical active fewer days per week and were more often current smokers than their counterparts (Table 2).

**Table 2:** Drug prescription data and health behaviour for patients at inclusion according to mental health and socioeconomic position.

	Depressive symptoms		Anxiety symptoms		Educational level			Cohabitation status	
	HADS-D≥8	HADS-D<8	HADS-A≥8	HADS-A<8	<10 years	10–12 years	>12 years	Living alone	Married/ cohabiting
Aspirin, %	77.3	78.9	79.2	78.4	79.9	79.0	72.7	79.1	78.4
Clopidogrel, %	74.3	77.5	77.7	76.8	74.4	79.6	79.5	72.8	78.1
β-Blocker, %	82.0	80.6	80.6	80.9	78.14	84.5	78.6	77.7	82.5
Statin, %	83.2	83.3	83.4	83.4	79.9*	87.6	85.5	77.0*	86.2
ACE-inhibitors/AT-II-receptor block, %	55.1	46.7	48.8	48.0	51.5	48.3	42.7	51.6	47.2
Antidepressants, %	22.2*	8.4	19.0*	8.5	11.3	11.6	9.4	15.0*	9.5
Secondary prophylactic medication**, %	65.9*	73.7	69.7	73.0	71.9	69.6	78.6	69.7	73.1
Physical activity, days/week, mean	3.7*	5.2	4.2*	5.2	4.8	4.9	5.3	4.7	5.0
Current smokers, %	25.9*	18.7	26.2*	18.2	21.5	18.7	16.4	23.0	18.5

*p<0.05

**We defined the participant as ‘receiving secondary prophylactic medication’ if the participant was receiving treatment with three or more of the following drugs: aspirin, clopidogrel, statins and β-blockers.

## Discussion

In this population-based cohort study, we found that post-MI patients living alone, with short and medium education and with depressive symptoms had a number of healthcare contacts that was similar to or lower than that of patients without these risk factors. On the other hand, MI patients with anxiety symptoms had a higher use of GP, but a lower use of hospital than patients without anxiety symptoms.

No previous studies have examined MI patients’ long-term overall use of the healthcare system (GP and hospital) according to mental health and socioeconomic position. However, previous studies of short-term hospital-based rehabilitation have also found a lower level of participation among MI patients living alone, with short education and with depressive symptoms than among MI patients without these risk factors. [15–18]

Low socioeconomic position [4–6] and depressive symptoms [1,2] after MI have consistently been associated with mortality, whereas anxiety symptoms have not. [21,38,39] Part of this difference in prognosis may be explained by differences in the use of healthcare. Patients with low socioeconomic position and depressive symptoms would be expected to have a higher need of healthcare contacts after the MI as they have a higher prevalence of risk factors and a poorer prognosis. Therefore even a similar level of healthcare contacts between patients with and without these risk factors may be alarming. Our and previous [15–18] studies indicate that patients with low socioeconomic position and depressive symptoms does not have a higher level of healthcare contacts after MI than patients without these risk factors, and several possible explanations for this have been suggested. Compared with patients with a long education, patients with a short education may have lower health literacy and lower awareness of physician diagnoses and recommendations. This may result in lower recognition of cardiac symptoms, fewer contacts to the healthcare system and less adherence to recommended lifestyle and medication. [16,40] Patients living with a partner may display less distress than their single-living counterparts as a partner can share the emotional burden and provide appropriate social support. A partner may also encourage the patient to seek medical help, attend cardiac rehabilitation and be adherent to secondary prophylactic medication. [41] It has previously been hypothesised that patients with anxiety may have a ‘constructive worrying’ capacity and therefore be more likely to seek help in response to less severe somatic symptoms, attend cardiac rehabilitation and be more adherent to secondary prophylactic medication, [42–44] whereas the opposite may be true for MI patients with depression. [7] Our results support this hypothesis as patients with anxiety symptoms had significantly more GP contacts after the MI than patients without anxiety symptoms. In contrast, patients with depressive symptoms only had the same number of GP contacts as patients without depressive symptoms. This underlines the importance of identification of depression as a part of routine care after MI, which is, regrettably, rarely performed. [20]

Contacts to the healthcare system are a requisite for rehabilitation after MI. More healthcare contacts may increase compliance and persistence to recommended lifestyle such as smoking cessation and regular physical activity. Regular evaluation of the medication may increase medication compliance, increase the probability of optimal secondary prophylactic treatment and potential drug side effects can be taken care of. Healthcare contacts are a requisite for a fast and correct diagnosis and thereby treatment of any new cardiovascular events. More healthcare contacts for patients with low socioeconomic position may improve their understanding of their disease and thereby improve their compliance and recognition of new cardiac symptoms. Healthcare contacts are also a requisite for diagnostics and treatment of a potential depression or anxiety disorder.

Physical activity among patients with depressive and anxiety symptoms may need extra attention, as these patients were less physical active than patients without these mental health problems in our study. Regarding secondary prophylactic medication, especially patients with depressive symptoms may need extra attention, whereas particularly statin treatment may need extra attention among patients with short education and patients living alone. Smoking cessation may also need extra attention, especially among patients with depressive and anxiety symptoms, as they were more often current smokers compared to patients without mental health problems. Only 22.2% of the patients with depressive symptoms were treated with antidepressants, this could indicate a gap in the diagnostics and treatment of patients with depression.

More contacts to the healthcare system among MI patients with depressive symptoms and low socioeconomic position may lead to better risk factor management, a more optimised medical treatment, less severe cardiac disease and lower mortality. This may be achieved through differentiated and outreaching treatment strategies for patients with these risk factors. A study on the short-term hospital-based rehabilitation has shown that social inequality in referral, attendance and adherence to rehabilitation can be remedied by systematic referral and a socially differentiated, individualised approach, [45] and that an extended rehabilitation program for socially vulnerable patients can increase the share of patients achieving treatment goals. [46] Katon et al. [47] conducted a study on collaborative care in a GP setting for patients with depression and diabetes and/or coronary heart disease. They found that compared with usual care, an intervention involving nurses who provided guideline-based, patient-centred management of depression and chronic disease significantly improved control of medical disease, depression, quality of life and satisfaction with care. Similarly, Richards et al. [48] found that collaborative care had persistent positive effects on depression up to 12 months after care initiation and was preferred by patients over usual care. These studies indicate that a systematic, stratified and outreaching approach may be the road ahead both in hospital and GP rehabilitation care. However, further studies are needed to examine if these interventions can also improve prognosis.

### Strengths and limitations of the study

The major strengths of this study are its population-based nature and its homogeneous study population; we invited all patients discharged with first-time MI during one year in a well-defined area. Our response rate was reasonably high (70.5%), and information on outcome was collected without loss to follow-up. Non-participants were more likely to be women, older, have fewer socioeconomic resources and a comorbid condition. In order to address the potential risk of selection bias, we estimated unadjusted GP contact rates for all the patients discharged with MI according to the available patient characteristics (cohabitation status, n = 1,671, and education, n = 1,513). In general, the patients with socioeconomic risk factors had more GP contacts than patients without these risk factors, but these trends tended to be smaller than the corresponding estimates for the participants. Thus, our results may overestimate the use of GP among patients with risk factors compared with patients without risk factors (S1 and S2 Figs).

Information on MI was registered prospectively and did not rely on the patients’ memory. The MI diagnosis was based on the current European Society of Cardiology criteria, coded by the physician in charge of the discharge, and is known to have a high sensitivity (90%) and specificity (92%). [49] The specificity was even higher in our study because we confirmed the MI diagnosis by reviewing the discharge summaries. We also reduced the risk of information bias by using previously translated and validated scales, by pilot-testing the questionnaire among MI patients, and by using high-quality register data. A diagnosis of depression or anxiety should ideally be based on a diagnostic interview. Since a previous study has estimated the sensitivity of the HADS-D≥8 for identification of depression to be 65% in MI patients, [31] a substantial number of participants with depression may have been misclassified as not having depression. However, we identified 18.6% with depressive symptoms in our population, which is in keeping with the prevalence of post-MI depression identified by structured clinical interviews in other studies (19.8%). [50] We found no studies reporting on the sensitivity and specificity of HADS-A in an MI population. However, among patients with acute coronary syndrome, a HADS-A≥8 had a sensitivity of 91%. [32] Accordingly, we most likely identified the majority of patients with anxiety symptoms. We evaluated depressive and anxiety symptoms 3 months after MI. This allowed the participants to naturally overcome depressive or anxiety symptoms after a stressful life event. We had information only on the number of healthcare contacts, but not on the reason for encounter or the contents of the contacts.

We accounted for the effect of confounding by depressive symptoms, anxiety symptoms, educational level, cohabitation status, dyspnoea score, comorbidity, sex and age. However, we cannot rule out the possibility of residual confounding.

### Conclusions

Patients with depressive symptoms, with short and medium education and patients living alone may have a lower long-term use of healthcare following MI than patients without these risk factors. Patients with depressive symptoms and low socioeconomic position would be expected to have a higher need of healthcare after MI as they have a poorer prognosis. A systematic, stratified and outreaching approach in the rehabilitation care may improve the quality of care among MI patients with mental health and socioeconomic risk factors.

## Supporting Information

S1 Fig Use of general practice before and after first-time MI per 3-month period according to educational level. Unadjusted contact rates (with 95% CI) for all patients discharged with MI and for participants. The date of MI is contained in the first period after the MI diagnosis.
(EPS)

S2 Fig Use of general practice before and after first-time MI per 3-month period according to cohabitation status. Unadjusted contact rates (with 95% CI) for all patients discharged with MI and for participants. The date of MI is contained in the first period after the MI diagnosis.
(EPS)

## References

[1] Meijer A, Conradi HJ, Bos EH, Anselmino M, Carney RM, Denollet J, et al Adjusted prognostic association of depression following myocardial infarction with mortality and cardiovascular events: individual patient data meta-analysis. Br J Psychiatry 2013 8;203(2):90–102. 10.1192/bjp.bp.112.111195 23908341

[2] Larsen KK, Christensen B, Sondergaard J, Vestergaard M. Depressive symptoms and risk of new cardiovascular events or death in patients with myocardial infarction: a population-based longitudinal study examining health behaviors and health care interventions. PLoS One 2013 9 25;8(9):e74393 10.1371/journal.pone.0074393 24086339 PMC3783427

[3] Nielsen TJ, Vestergaard M, Christensen B, Christensen KS, Larsen KK. Mental health status and risk of new cardiovascular events or death in patients with myocardial infarction: a population-based cohort study. BMJ Open 2013 8 2;3(8): 10.1136/bmjopen-2013-003045 PMC3733312 23913773

[4] Case RB, Moss AJ, Case N, McDermott M, Eberly S. Living alone after myocardial infarction. Impact on prognosis. JAMA 1992 1 22–29;267(4):515–519. 1729573

[5] Rasmussen JN, Rasmussen S, Gislason GH, Buch P, Abildstrom SZ, Kober L, et al Mortality after acute myocardial infarction according to income and education. J Epidemiol Community Health 2006 4;60(4):351–356. 16537354 10.1136/jech.200X.040972 PMC2566173

[6] Tonne C, Schwartz J, Mittleman M, Melly S, Suh H, Goldberg R. Long-term survival after acute myocardial infarction is lower in more deprived neighborhoods. Circulation 2005 6 14;111(23):3063–3070. 15939820 10.1161/CIRCULATIONAHA.104.496174

[7] Carney RM, Freedland KE, Miller GE, Jaffe AS. Depression as a risk factor for cardiac mortality and morbidity: a review of potential mechanisms. J Psychosom Res 2002 10;53(4):897–902. 12377300 10.1016/s0022-3999(02)00311-2

[8] Huffman JC, Celano CM, Januzzi JL. The relationship between depression, anxiety, and cardiovascular outcomes in patients with acute coronary syndromes. Neuropsychiatr Dis Treat 2010 5 6;6:123–136. 20505844 10.2147/ndt.s6880 PMC2874336

[9] Ziegelstein RC, Fauerbach JA, Stevens SS, Romanelli J, Richter DP, Bush DE. Patients with depression are less likely to follow recommendations to reduce cardiac risk during recovery from a myocardial infarction. Arch Intern Med 2000 6 26;160(12):1818–1823. 10871976 10.1001/archinte.160.12.1818

[10] Murphy BM, Grande MR, Navaratnam HS, Higgins RO, Elliott PC, Turner A, et al Are poor health behaviours in anxious and depressed cardiac patients explained by sociodemographic factors? Eur J Prev Cardiol 2013 12;20(6):995–1003. 10.1177/2047487312449593 22626910

[11] Mayer O Jr, Simon J, Heidrich J, Cokkinos DV, De Bacquer D, EUROASPIRE II Study Group. Educational level and risk profile of cardiac patients in the EUROASPIRE II substudy. J Epidemiol Community Health 2004 1;58(1):47–52. 14684726 10.1136/jech.58.1.47 PMC1757031

[12] Green P, Newman JD, Shaffer JA, Davidson KW, Maurer MS, Schwartz JE. Relation of patients living without a partner or spouse to being physically active after acute coronary syndromes (from the PULSE accelerometry substudy). Am J Cardiol 2013 5 1;111(9):1264–1269. 10.1016/j.amjcard.2013.01.272 23411104 PMC3640672

[13] Heran BS, Chen JM, Ebrahim S, Moxham T, Oldridge N, Rees K, et al Exercise-based cardiac rehabilitation for coronary heart disease. Cochrane Database Syst Rev 2011 7 6;(7):CD001800. doi(7):CD001800. 10.1002/14651858.CD001800.pub2 21735386 PMC4229995

[14] Taylor RS, Brown A, Ebrahim S, Jolliffe J, Noorani H, Rees K, et al Exercise-based rehabilitation for patients with coronary heart disease: systematic review and meta-analysis of randomized controlled trials. Am J Med 2004 5 15;116(10):682–692. 15121495 10.1016/j.amjmed.2004.01.009

[15] Jackson L, Leclerc J, Erskine Y, Linden W. Getting the most out of cardiac rehabilitation: a review of referral and adherence predictors. Heart 2005 1;91(1):10–14. 15604322 10.1136/hrt.2004.045559 PMC1768637

[16] Alter DA, Iron K, Austin PC, Naylor CD, SESAMI Study Group. Socioeconomic status, service patterns, and perceptions of care among survivors of acute myocardial infarction in Canada. JAMA 2004 3 3;291(9):1100–1107. 14996779 10.1001/jama.291.9.1100

[17] Nielsen KM, Faergeman O, Foldspang A, Larsen ML. Cardiac rehabilitation: health characteristics and socio-economic status among those who do not attend. Eur J Public Health 2008 10;18(5):479–483. 10.1093/eurpub/ckn060 18614608

[18] Glazer KM, Emery CF, Frid DJ, Banyasz RE. Psychological predictors of adherence and outcomes among patients in cardiac rehabilitation. J Cardiopulm Rehabil 2002 Jan-Feb;22(1):40–46. 11839996 10.1097/00008483-200201000-00006

[19] Larsen KK, Vestergaard M, Sondergaard J, Christensen B. Rehabilitation status three months after first-time myocardial infarction. Scand J Prim Health Care 2011 12;29(4):210–215. 10.3109/02813432.2011.629147 22126219 PMC3308468

[20] Larsen KK, Vestergaard M, Sondergaard J, Christensen B. Screening for depression in patients with myocardial infarction by general practitioners. Eur J Prev Cardiol 2012 4 10. 10.1177/2047487312444994 22496274

[21] Larsen KK, Christensen B, Nielsen TJ, Vestergaard M. Post-myocardial infarction anxiety or depressive symptoms and risk of new cardiovascular events or death: a population-based longitudinal study. Psychosom Med 2014 Nov-Dec;76(9):739–746. 10.1097/PSY.0000000000000115 25373894

[22] Andersen TF, Madsen M, Jorgensen J, Mellemkjoer L, Olsen JH. The Danish National Hospital Register. A valuable source of data for modern health sciences. Dan Med Bull 1999 6;46(3):263–268. 10421985

[23] Pedersen CB. The Danish Civil Registration System. Scand J Public Health 2011 7;39(7 Suppl):22–25. 10.1177/1403494810387965 21775345

[24] Pedersen KM, Andersen JS, Sondergaard J. General practice and primary health care in Denmark. J Am Board Fam Med 2012 3;25 Suppl 1:S34–8. 10.3122/jabfm.2012.02.110216 22403249

[25] Andersen JS, Olivarius Nde F, Krasnik A. The Danish National Health Service Register. Scand J Public Health 2011 7;39(7 Suppl):34–37. 10.1177/1403494810394718 21775348

[26] Statistics Denmark. IDA–an integrated database for labour market research: Main report. Copenhagen: Statistics Denmark. 1991.

[27] Zigmond AS, Snaith RP. The Hospital Anxiety and Depression Scale. Acta Psychiatr Scand 1983 1983;67(6). 10.1111/j.1600-0447.1983.tb09716.x 6880820

[28] Johnston M, Pollard B, Hennessey P. Construct validation of the hospital anxiety and depression scale with clinical populations. J Psychosom Res 2000 6;48(6):579–584. 11033377 10.1016/s0022-3999(00)00102-1

[29] Martin CR, Lewin RJ, Thompson DR. A confirmatory factor analysis of the Hospital Anxiety and Depression Scale in coronary care patients following acute myocardial infarction. Psychiatry Res 2003 8 30;120(1):85–94. 14500117 10.1016/s0165-1781(03)00162-8

[30] Bjelland I, Dahl AA, Haug TT, Neckelmann D. The validity of the Hospital Anxiety and Depression Scale. An updated literature review. J Psychosom Res 2002 2;52(2):69–77. 11832252 10.1016/s0022-3999(01)00296-3

[31] Thombs BD, Magyar-Russell G, Bass EB, Stewart KJ, Tsilidis KK, Bush DE, et al Performance characteristics of depression screening instruments in survivors of acute myocardial infarction: review of the evidence. Psychosomatics 2007 May-Jun;48(3):185–194. 17478586 10.1176/appi.psy.48.3.185

[32] Frasure-Smith N, Lesperance F. Depression and anxiety as predictors of 2-year cardiac events in patients with stable coronary artery disease. Arch Gen Psychiatry 2008 1;65(1):62–71. 10.1001/archgenpsychiatry.2007.4 18180430

[33] Fletcher C. Standardized questionaires on respiratory symptoms. A statement prepared for, and approved by, the Medical Research Council's Committee on the aetiology of chronic bronchitis. Br Med J 1960;2:1665.13688719

[34] Vestbo J, Knudsen KM, Rasmussen FV. Should we Continue using Questionnaires on Breathlessness in Epidemiologic Surveys. Am Rev Respir Dis 1988 5 1988;137(5). 10.1164/ajrccm/137.5.1114 3195810

[35] Figarska SM, Boezen HM, Vonk JM. Dyspnea severity, changes in dyspnea status and mortality in the general population: the Vlagtwedde/Vlaardingen study. Eur J Epidemiol 2012 11;27(11):867–876. 10.1007/s10654-012-9736-0 23054033 PMC3501159

[36] Carstensen B, Kristensen JK, Marcussen MM, Borch-Johnsen K. The National Diabetes Register. Scand J Public Health 2011 7;39(7 Suppl):58–61. 10.1177/1403494811404278 21775353

[37] Johannesdottir SA, Horvath-Puho E, Ehrenstein V, Schmidt M, Pedersen L, Sorensen HT. Existing data sources for clinical epidemiology: The Danish National Database of Reimbursed Prescriptions. Clin Epidemiol 2012;4:303–313. 10.2147/CLEP.S37587 23204870 PMC3508607

[38] Doyle F, Conroy R, McGee H. Differential predictive value of depressive versus anxiety symptoms in the prediction of 8-year mortality after acute coronary syndrome. Psychosom Med 2012 9;74(7):711–716. 10.1097/PSY.0b013e318268978e 22923700

[39] Frasure-Smith N, Lesperance F. Depression and other psychological risks following myocardial infarction. Arch Gen Psychiatry 2003 6;60(6):627–636. 12796226 10.1001/archpsyc.60.6.627

[40] McKee MM, Winters PC, Fiscella K. Low education as a risk factor for undiagnosed angina. J Am Board Fam Med 2012 Jul-Aug;25(4):416–421. 10.3122/jabfm.2012.04.110282 22773709 PMC3607287

[41] Aizer AA, Chen MH, McCarthy EP, Mendu ML, Koo S, Wilhite TJ, et al Marital status and survival in patients with cancer. J Clin Oncol 2013 11 1;31(31):3869–3876. 10.1200/JCO.2013.49.6489 24062405 PMC4878087

[42] Mykletun A, Bjerkeset O, Dewey M, Prince M, Overland S, Stewart R. Anxiety, depression, and cause-specific mortality: the HUNT study. Psychosom Med 2007 5;69(4):323–331. 17470669 10.1097/PSY.0b013e31803cb862

[43] Kim HK, Park JH, Park JH, Kim JH. Differences in adherence to antihypertensive medication regimens according to psychiatric diagnosis: results of a Korean population-based study. Psychosom Med 2010 1;72(1):80–87. 10.1097/PSY.0b013e3181c4e3e9 19933508

[44] Grace SL, Abbey SE, Shnek ZM, Irvine J, Franche RL, Stewart DE. Cardiac rehabilitation II: referral and participation. Gen Hosp Psychiatry 2002 May-Jun;24(3):127–134. 12062136 10.1016/s0163-8343(02)00179-2

[45] Meillier LK, Nielsen KM, Larsen FB, Larsen ML. Socially differentiated cardiac rehabilitation: can we improve referral, attendance and adherence among patients with first myocardial infarction? Scand J Public Health 2012 5;40(3):286–293. 10.1177/1403494812443600 22637368

[46] Nielsen KM, Meillier LK, Larsen ML. Extended cardiac rehabilitation for socially vulnerable patients improves attendance and outcome. Dan Med J 2013 3;60(3):A4591 23484610

[47] Katon WJ, Lin EH, Von Korff M, Ciechanowski P, Ludman EJ, Young B, et al Collaborative care for patients with depression and chronic illnesses. N Engl J Med 2010 12 30;363(27):2611–2620. 10.1056/NEJMoa1003955 21190455 PMC3312811

[48] Richards DA, Hill JJ, Gask L, Lovell K, Chew-Graham C, Bower P, et al Clinical effectiveness of collaborative care for depression in UK primary care (CADET): cluster randomised controlled trial. BMJ 2013 8 19;347:f4913 10.1136/bmj.f4913 23959152 PMC3746956

[49] Joensen AM, Jensen MK, Overvad K, Dethlefsen C, Schmidt E, Rasmussen L, et al Predictive values of acute coronary syndrome discharge diagnoses differed in the Danish National Patient Registry. J Clin Epidemiol 2009 2;62(2):188–194. 10.1016/j.jclinepi.2008.03.005 18722087

[50] Thombs BD, Bass EB, Ford DE, Stewart KJ, Tsilidis KK, Patel U, et al Prevalence of depression in survivors of acute myocardial infarction—Review of the evidence. Journal of General Internal Medicine 2006 1 2006;21(1). 10.1111/j.1525-1497.2005.00269.x PMC1484630 16423120

